# Single-dose fosaprepitant for the prevention of chemotherapy-induced nausea and vomiting in patients receiving moderately emetogenic chemotherapy regimens: a subgroup analysis from a randomized clinical trial of response in subjects by cancer type

**DOI:** 10.1186/s12885-020-07259-5

**Published:** 2020-09-25

**Authors:** Cindy Weinstein, Karin Jordan, Stuart Green, Saleem Khanani, Elizabeth Beckford-Brathwaite, Waldimir Vallejos, Annpey Pong, Stephen J. Noga, Bernardo L. Rapoport

**Affiliations:** 1 Merck & Co., Inc., 2000 Galloping Hill Road, Kenilworth, NJ USA; 2 University of Heidelberg, Im Neuenheimer Feld 400, 69120 Heidelberg, Germany; 3 Heywood Hospital, 242 Green Street, Gardner, MA USA; 4 Weinberg Cancer Institute, 9103 Franklin Square Drive, Baltimore, MD USA; 5 Department of Immunology, Faculty of Health Sciences, University of Pretoria, Corner Doctor Savage Road and Bophelo Road, Pretoria, 0002 South Africa; 6 The Medical Oncology Centre of Rosebank, 129 Oxford Road, Saxonwold, Johannesburg, 2196 South Africa

**Keywords:** Chemotherapy-induced nausea and vomiting (CINV), Fosaprepitant, Gastrointestinal cancer, Lung cancer, Breast cancer, Gynecologic cancer

## Abstract

**Background:**

Results from a phase III, randomized, double-blind, active comparator-controlled, parallel-group trial evaluating fosaprepitant for the prevention of chemotherapy-induced nausea and vomiting (CINV) found that a single-day, triple-antiemetic fosaprepitant regimen resulted in a significantly higher proportion of patients achieving a complete response (CR; no vomiting or rescue medication use) in the delayed phase (25–120 h after chemotherapy initiation), compared with a 3-day control regimen (ClinicalTrials.gov, NCT01594749). As the risk for CINV is dependent on chemotherapy regimen and generally guided by tumor type, this post hoc analysis evaluated the efficacy and safety of this regimen by cancer subpopulations (gastrointestinal [GI] or colorectal, lung, breast, and gynecologic cancers).

**Methods:**

Subjects with confirmed cancer who were naive to highly and moderately emetogenic chemotherapy (HEC and MEC) and were scheduled to receive intravenous (IV) anthracycline-cyclophosphamide (AC)–based MEC on the first day of chemotherapy were randomly assigned to receive oral ondansetron and oral dexamethasone plus either a single IV dose of fosaprepitant 150 mg (fosaprepitant regimen) or placebo (control regimen). The primary efficacy end point was the proportion of subjects achieving CR in the delayed phase. CR rates in the overall and acute phases (0–120 h and 0–24 h after MEC initiation, respectively) were assessed as secondary end points. Safety and tolerability were also assessed.

**Results:**

CR rates in the delayed phase favored the fosaprepitant regimen over the control regimen across the GI/colorectal, lung, breast, and gynecologic cancer subgroups (range, 6.2–22%); similar findings were observed for CR in the overall phase. CR in the acute phase was high for all groups (≥87%). The fosaprepitant regimen was well tolerated in all cancer subgroups.

**Conclusions:**

This post hoc analysis indicated that a single-day fosaprepitant regimen was effective in preventing CINV in patients receiving MEC, regardless of cancer type.

**Trial registration:**

ClinicalTrials.govNCT01594749, registered May 9, 2012.

## Background

The prevention of chemotherapy-induced nausea and vomiting (CINV), a common and potentially treatment-limiting side effect of cancer therapy, remains an important challenge [1–3]. Since CINV risk factors depend mainly on chemotherapy regimen, which is generally guided by tumor type (e.g., National Comprehensive Cancer Network cancer treatment regimens), antiemetic requirements may differ across tumor types. The emetic risk of agents considered to be moderately emetogenic chemotherapy (MEC) ranges from 30 to 90%, and the risk for those considered to be highly emetogenic chemotherapy (HEC) is > 90% [4–6]. Estimating the emetic risk of multiagent chemotherapy regimens is more challenging; tools have been devised to estimate the emetogenicity of multiagent chemotherapy regimens [7, 8]. Current guidelines recommend prescribing antiemetics based on the chemotherapy agent with the highest emetogenic risk [6].

Neurokinin-1 receptor antagonists have been extensively studied and incorporated into treatment guidelines in various combinations with other antiemetic agents for prevention of CINV from MEC therapy [4, 6, 9]. The National Comprehensive Cancer Network recommends this drug class for MEC-induced CINV generally, and specific recommendations in other guidelines include adults treated with carboplatin (drug class) [4, 9] and children who are unable to tolerate dexamethasone (aprepitant) [9]. These recommendations have been supported by multiple phase III trials, including the study of fosaprepitant we present in this article [4, 6, 9].

Fosaprepitant is a neurokinin-1 receptor antagonist that is indicated in adults and children aged 6 months and older, in combination with other antiemetic agents, for the prevention of acute and delayed nausea and vomiting associated with initial and repeat courses of HEC and delayed nausea and vomiting associated with initial and repeat courses of MEC [10].

PN031 was a phase III, randomized, double-blind, active comparator-controlled, parallel-group trial that investigated the efficacy and safety of a single-dose, triple-antiemetic, fosaprepitant regimen for the prevention of CINV in patients (*N* = 1000) receiving non–anthracycline-cyclophosphamide (AC)-based MEC with any type of malignancy [11]. AC regimens, although traditionally considered MEC, were reclassified as HEC in 2011 because of their propensity to induce CINV, particularly nausea, in patients with breast cancer [12–14] and were thus excluded. The primary end point of PN031 was met, with the single-day fosaprepitant regimen demonstrating a significant improvement in the proportion of patients achieving a CR (no vomiting or rescue medication use) in the delayed phase (25–120 h after MEC initiation), compared with the 3-day control regimen [11].

Because chemotherapy regimens and emetogenic risk vary based on the type of cancer, it is important to evaluate the ability of antiemetic regimens to prevent CINV in different patient populations. Herein, we report the results of a post hoc analysis of PN031, which evaluated the efficacy of a single-day fosaprepitant regimen compared with a standard 3-day control regimen in the most common cancer subpopulations in the study (ie, gastrointestinal [GI] or colorectal, lung, breast, and gynecologic).

## Methods

### Study design and population

The current report is a post hoc analysis based on data from a phase III, multicenter, global, double-blind, randomized trial (PN031) [11]. Details of the study entry criteria have been published previously [11] (ClinicalTrials.gov identifier: NCT01594749).

In brief, adult subjects with histologically or cytologically confirmed cancer who were naive to HEC and MEC and were scheduled to receive ≥1 IV dose of non-AC MEC on day 1 were included [11]. Major exclusion criteria included the following: vomiting in the 24-h period before day 1; symptomatic primary or metastatic central nervous system malignancy causing nausea and/or vomiting; and use of any dose of cisplatin or other HEC [11].

The trial adhered to the International Conference on Harmonization Good Clinical Practice guidelines and was conducted in agreement with the Declaration of Helsinki. The protocol was approved by health authorities and ethics committees/institutional review boards prior to study initiation for all participating study centers. All subjects were required to provide written informed consent before study enrollment.

### Study treatments

Study treatments have been described in detail previously [11] and are summarized here. Subjects were randomly assigned 1:1 to a single IV dose of fosaprepitant 150 mg (fosaprepitant regimen) or placebo (control regimen), administered approximately 30 min before MEC initiation on day 1. Both treatment groups received oral ondansetron plus oral dexamethasone on day 1 before MEC, followed by oral ondansetron 8 h later. Subjects in the control group also received ondansetron every 12 h on days 2 and 3; the fosaprepitant group received matching placebo on these days. Investigator-prescribed rescue medication was permitted throughout the study.

### Study outcomes

The proportion of subjects who achieved CR during the delayed phase served as the primary efficacy end point. Secondary efficacy end points included: the proportion of subjects achieving CR during the overall (0–120 h after MEC initiation) and acute phases (0–24 h after MEC initiation); and the proportion of subjects with no vomiting (no emetic episodes, including no vomit [expulsion of stomach contents through the mouth] and no retching or dry heaves [an attempt to vomit that is not productive of stomach contents], regardless of use of rescue medication) during the overall phase.

National Cancer Institute Common Terminology Criteria for Adverse Events, version 4.0 was used to assess adverse events (AEs). Events that the investigator considered to be related to any of the study medications were recorded as treatment-related AEs.

### Statistical analysis

Efficacy analyses included participants who received ≥1 dose of study drug and in accordance with the intention-to-treat (ITT) principle were analyzed in their randomly assigned treatment group. Safety analyses following the all-subjects-as-treated (ASaT) approach included participants who received ≥1 dose of study drug and who were analyzed in the treatment group based on the drug actually received. The primary and secondary efficacy end points were explored for the ITT population in the most commonly reported cancer subpopulations for PN031, which were GI or colorectal, lung, breast, and gynecologic cancer. Treatment group comparisons were made using the Cochran-Mantel-Haenszel test [15] (stratified by sex) and 95% confidence intervals (CIs) calculated by the Miettinen-Nurminen method [16]. Time-to-first vomiting episode was analyzed using the Kaplan-Meier method [17]. Demographic variables, baseline characteristics, and AEs were summarized with descriptive statistics.

With a sample size of 80 subjects from each subgroup, the power to detect the 20% difference in complete response between groups is 73% with a 5% two-sided significance level. In other words, the sample sizes are adequate for some group comparisons. For smaller subgroups by chemotherapy, there was no formal testing. This post hoc presentation is provided for descriptive purposes only.

## Results

### Subjects

The overall ITT population included 1000 subjects, which comprised 502 subjects assigned to the fosaprepitant regimen and 498 assigned to the control regimen. The most common cancer subgroups included (ASaT population) GI or colorectal cancer (*n* = 267; fosaprepitant regimen, *n* = 135, and control regimen, *n* = 132); lung cancer (*n* = 254; fosaprepitant regimen, *n* = 130, and control regimen, *n* = 124); breast cancer (*n* = 231; fosaprepitant regimen, *n* = 110, and control regimen, *n* = 121); and gynecologic cancer (*n* = 152; fosaprepitant regimen, *n* = 81, and control regimen, *n* = 71). The following cancer subgroups had too few patients for analysis: head and neck cancer (*n* = 21; fosaprepitant regimen, *n* = 12; control regimen, *n* = 9), germ cell cancer (*n* = 2; fosaprepitant regimen, *n* = 0; control regimen, *n* = 2), hepatobiliary cancer (*n* = 8; fosaprepitant regimen, *n* = 2; control regimen, *n* = 6), lymphoproliferative cancer (*n* = 8; fosaprepitant regimen, *n* = 4; control regimen, *n* = 4), sarcoma (*n* = 9; fosaprepitant regimen, *n* = 5; control regimen, *n* = 4), skin cancer (*n* = 3; fosaprepitant regimen, *n* = 1; control regimen, *n* = 2), and unspecified other types (*n* = 45; fosaprepitant regimen, *n* = 23; control regimen, *n* = 22). Baseline characteristics and CINV prognostic factors were generally well balanced between the treatment groups within cancer subgroups (Table 1).

**Table 1:** Demographics and baseline characteristics (ASaT population)

	Fosaprepitant regimen				Control regimen			
	GI or colorectal cancers (*n* = 135)	Lung cancer (*n* = 130)	Breast cancer (*n* = 110)	Gynecologic cancer (*n* = 81)	GI or colorectal cancers (*n* = 132)	Lung cancer (*n* = 124)	Breast cancer (*n* = 121)	Gynecologic cancer (*n* = 71)
Age, years								
Median (range)	62 (24–85)	65 (36–82)	57 (28–78)	58 (31–88)	60 (23–88)	65 (38–82)	54 (28–81)	56 (24–78)
Age group, *n* (%)								
< 50 years	21 (15.6)	7 (5.4)	37 (33.6)	25 (30.9)	21 (15.9)	5 (4.0)	39 (32.2)	24 (33.8)
≥ 50 years	114 (84.4)	123 (94.6)	73 (66.4)	56 (69.1)	111 (84.1)	119 (96.0)	82 (67.8)	47 (66.2)
Sex, *n* (%)								
Male	86 (63.7)	90 (69.2)	1 (0.9)	0	76 (57.6)	90 (72.4)	0	0
Female	49 (36.3)	40 (30.8)	109 (99.1)	81 (100)	56 (42.4)	34 (27.4)	121 (100)	71 (100)
History of motion sickness, *n* (%)	8 (5.9)	5 (3.8)	5 (4.5)	8 (9.9)	3 (2.3)	4 (3.2)	12 (9.9)	9 (12.7)
History of emesis during pregnancy, *n* (%)	11 (8.1)	7 (5.4)	24 (21.8)	15 (18.5)	15 (11.4)	6 (4.8)	23 (19.0)	15 (21.1)
History of alcohol use, *n* (%)	69 (51.1)	68 (52.3)	35 (31.8)	30 (37.0)	58 (43.9)	67 (54.0)	41 (33.9)	21 (29.6)
Chemotherapy regimen, *n* (%)^a^	*n* = 135	*n* = 129	*n* = 110	*n* = 81	*n* = 132	*n* = 125	*n* = 121	*n* = 71
Single day	28 (20.7)	108 (83.7)	100 (90.9)	81 (100)	21 (15.9)	109 (87.2)	110 (90.9)	69 (97.2)
Multiple day	105 (77.8)	18 (14.0)	0	0	109 (82.6)	14 (11.2)	0	2 (2.8)

*ASaT* all-subjects-as-treated, *GI* gastrointestinal

^a^Based on the intention-to-treat population

### Cancer subgroup chemotherapy regimens

Chemotherapy regimens that were used across the analyzed cancer subgroups are summarized in Table 2.

**Table 2:** Subject characteristics by chemotherapy categories (ITT population)

	Fosaprepitant regimen				Control regimen			
*n* (%)	GI or colorectal cancers (*n* = 135)	Lung cancer (*n* = 129)	Breast cancer (*n* = 110)	Gynecologic cancer (*n* = 81)	GI or colorectal cancers (*n* = 132)	Lung cancer (*n* = 125)	Breast cancer (*n* = 121)	Gynecologic cancer (*n* = 71)
**Single-day regimens**								
Single MEC	4 (3.0)	12 (9.3)	10 (9.1)	9 (11.1)	4 (3.0)	21 (16.8)	3 (2.5)	7 (9.9)
MEC + ≥1 LEC	24 (17.8)	96 (74.4)	90 (81.8)	72 (89)	16 (12.1)	88 (70.4)	106 (87.6)	61 (85.9)
MEC + MEC	0	0	0	0	1 (0.8)	0	1 (0.8)	1 (1.4)
**Multiple-day regimens**								
MEC (day 1) + ≥1 LEC beyond day 1	100 (74.1)	18 (14.0)	0	0	94 (71.2)	14 (11.2)	0	2 (2.8)
MEC (day 1) + ≥1 MEC + ≥1 LEC	5 (3.7)	0	0	0	15 (11.4)	0	0	0
**Non-MEC regimens or no MEC on day 1**								
LEC only	2 (1.5)	0	8 (7.3)	0	2 (1.5)	1 (0.8)	8 (6.6)	0
Chemotherapy on day 2 only^a^	0	0	0	0	0	0	0	0
HEC regimens	0	3 (2.3)	2 (1.8)	0	0	1 (0.8)	3 (2.5)	0
Cisplatin-based	–	3 (2.3)	–	–	–	1 (0.8)	–	–
AC-based	–	–	2 (1.8)	–	–	–	3 (2.5)	–

*AC* anthracycline + cyclophosphamide, *HEC* highly emetogenic chemotherapy, *ITT* intent to treat, *LEC* low emetogenic chemotherapy, *MEC* moderately emetogenic chemotherapy

^a^Subject received MEC + LEC on day 2

In the GI or colorectal cancer subgroup, the majority of subjects received multiple-day chemotherapy regimens in the fosaprepitant and control regimen treatment arms (77.8 and 82.6%, respectively); single-day regimens were used by 20.7 and 15.9% of subjects, respectively. Most subjects in this cancer subgroup received non–carboplatin-based MEC; only 5 subjects in each treatment arm received carboplatin-based chemotherapy. Within the non–carboplatin-based MEC regimens, oxaliplatin or irinotecan alone or in combination with other antineoplastic agents (of a heterogenous emetogenic potential) were received by all but 5 subjects (3 in the fosaprepitant arm, 2 in the control arm).

In the lung cancer subgroup, most subjects received single-day MEC regimens, comprising primarily MEC plus one or more low emetogenic chemotherapy (LEC) agent(s) (74.4 and 70.4% subjects for the fosaprepitant and control regimens, respectively). Multiple-day regimens were received by 18 (14.0%) and 14 (11.2%) subjects, respectively. Nearly all participants (98%), regardless of chemotherapy duration, received a carboplatin-containing regimen.

In the breast cancer subgroup, most subjects received single-day MEC regimens (90.9% in both treatment groups); overall, 84.8% received a MEC plus one or more LEC regimen. No subjects received multiple-day chemotherapy regimens. The most commonly used regimens were those containing cyclophosphamide (47.6% received cyclophosphamide with a LEC or MEC agent).

In the gynecologic cancer subgroup, all but 2 subjects (both in the control regimen) received single-day MEC regimens. Most subjects received carboplatin-based chemotherapy (93.8 and 93.0% of subjects in the fosaprepitant and control groups, respectively).

### Complete response rates by cancer subgroup

**Fig. 1:**
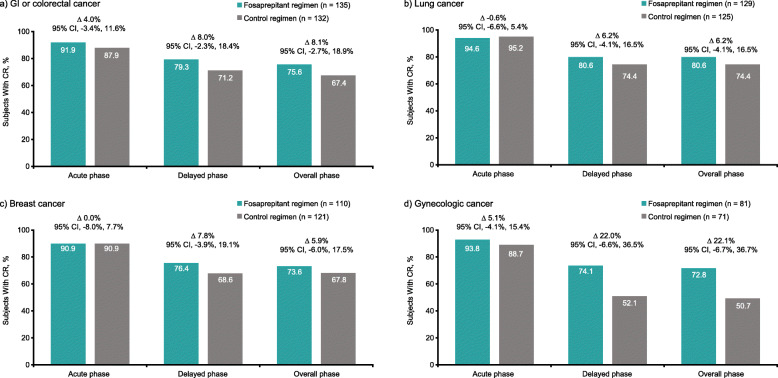
Proportion of subjects achieving a CR for the fosaprepitant regimen versus the control regimen: acute (0–24 h), delayed (25–120 h), and overall (0–120 h) CR rates based on the Cochran-Mantel-Haenszel method, with stratification by sex (ITT population). **a** Subjects with GI or colorectal cancer. **b** Subjects with lung cancer. **c** Subjects with breast cancer. **d** Subjects with gynecologic cancer. *CI* confidence interval, *CR* complete response, *GI* gastrointestinal, *ITT* intention-to-treat

The percentages of subjects achieving CR in the delayed phase were higher with the fosaprepitant regimen, compared with the control regimen across all cancer subgroups (Fig. 1a–d). Treatment differences in CR rates between the fosaprepitant and control regimens in the delayed phase were 8.0% for GI or colorectal cancers, 6.2% for lung cancer, 7.8% for breast cancer, and 22.0% for gynecologic cancer.

Similar findings were also observed for treatment differences in CR rates in the overall phase (8.1, 6.2, 5.9, and 22.1%, respectively), whereas CR rates in the acute phase were high (> 87%) in both treatment arms for all cancer subtypes with no notable treatment differences (4.0, − 0.6, 0.0, and 5.1%, respectively) (Fig. 1a–d).

### No vomiting episodes by cancer subgroups

**Fig. 2:**
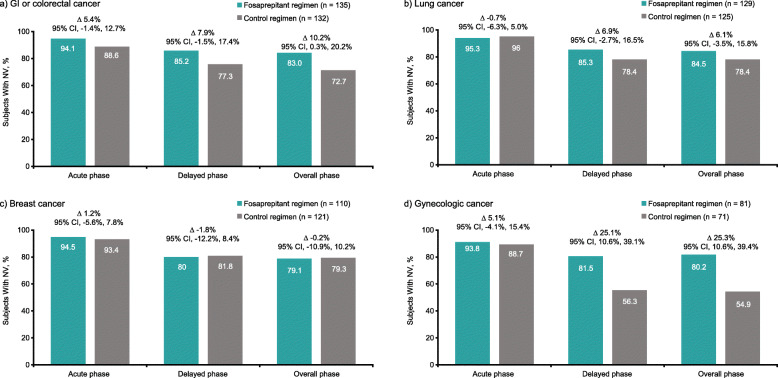
Proportion of subjects achieving no vomiting for the fosaprepitant regimen versus the control regimen: acute (0–24 h), delayed (25–120 h), and overall (0–120 h) no vomiting rates based on the Cochran-Mantel-Haenszel method, with stratification by sex (ITT population). **a** Subjects with GI or colorectal cancer. **b** Subjects with lung cancer. **c** Subjects with breast cancer. **d** Subjects with gynecologic cancer. *CI* confidence interval, *GI* gastrointestinal, *ITT* intention-to-treat, *NV* no vomiting

The percentages of subjects with no vomiting in the delayed, overall, and acute phases are summarized in Fig. 2a–d. In the GI or colorectal cancer subgroup, higher rates were observed with the fosaprepitant regimen versus the control regimen during all 3 phases; the greatest treatment difference between the fosaprepitant and control regimens was seen during the overall phase (10.2%). In the lung cancer subgroup, numerically higher rates were observed with the fosaprepitant regimen versus the control regimen during the delayed and overall phases; similar rates were seen between treatment groups in the acute phase. Similar rates between the fosaprepitant and control regimens were reported in all 3 phases for patients in the breast cancer subgroup. Finally, higher rates were observed with the fosaprepitant regimen versus the control regimen during all 3 phases in the gynecologic cancer subgroup.

### Time-to-first vomiting episode by cancer subgroups

**Fig. 3:**
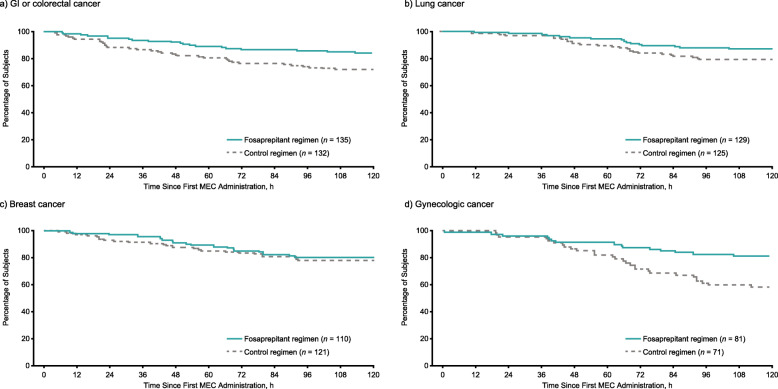
Kaplan-Meier curves of time to first vomiting episode from the first initiation of MEC for the fosaprepitant regimen versus the control regimen during the overall (0–120 h) phase (ITT population). **a** Subjects with GI or colorectal cancer. **b** Subjects with lung cancer. **c** Subjects with breast cancer. **d** Subjects with gynecologic cancer. *GI* gastrointestinal, *ITT* intent-to-treat, *MEC* moderately emetogenic chemotherapy

Kaplan-Meier analyses indicated that the time-to-first vomiting episode was delayed in the fosaprepitant regimen group, compared with the control regimen group in all cancer subgroups; however, differences in the subgroup of subjects with breast cancer were small (Fig. 3a–d).

### Safety by cancer subgroup

The fosaprepitant regimen was generally well tolerated across all cancer subgroups. The proportions of subjects with at least 1 AE and with at least 1 treatment-related AE by cancer subgroup were similar between the fosaprepitant and control regimens for all subgroups. AEs appeared to be well balanced between treatment groups across all cancer subgroups; the most common grade 1–4 AEs are summarized in Table 3. Diarrhea was the most commonly observed AE in the GI or colorectal cancer subgroups (18.5 and 17.4%), fatigue was the most commonly observed AE in subjects with lung cancer (13.1 and 9.7%) and gynecologic cancer (12.3 and 14.1%), for the fosaprepitant and control regimens, respectively. In the breast cancer subgroup, neutropenia, fatigue, and diarrhea were observed most frequently.

**Table 3:** Summary of the most common (≥5%) adverse events^a^ (ASaT population)

	Fosaprepitant regimen				Control regimen			
AE, *n* (%	GI or colorectal cancers (*n* = 135)	Lung cancer (*n* = 130)	Breast cancer (*n* = 110)	Gynecologic cancer (*n* = 81)	GI or colorectal cancers (*n* = 132)	Lung cancer (*n* = 124)	Breast cancer (*n* = 121)	Gynecologic cancer (*n* = 71)
Any	83 (61.5)	73 (56.2)	86 (78.2)	41 (50.6)	80 (60.6)	66 (53.2)	88 (72.7)	42 (59.2)
Neutropenia	2 (1.5)	7 (5.4)	27 (24.5)	3 (3.7)	1 (0.8)	5 (4.0)	23 (19.0)	5 (7.0)
Diarrhea	25 (18.5)	8 (6.2)	19 (17.3)	5 (6.2)	23 (17.4)	4 (3.2)	24 (19.8)	4 (5.6)
Fatigue	18 (13.3)	17 (13.1)	26 (23.6)	10 (12.3)	13 (9.8)	12 (9.7)	25 (20.7)	10 (14.1)
Constipation	18 (13.3)	7 (5.4)	12 (10.9)	8 (9.9)	10 (7.6)	10 (8.1)	20 (16.5)	8 (11.3)
Headache	11 (8.1)	4 (3.1)	12 (10.9)	2 (2.5)	12 (9.1)	3 (2.4)	16 (13.2)	2 (2.8)
Decreased appetite	11 (8.1)	5 (3.8)	5 (4.5)	5 (6.2)	11 (8.3)	7 (5.6)	10 (8.3)	3 (4.2)
Dysgeusia	4 (3.0)	3 (2.3)	9 (8.2)	2 (2.5)	7 (5.3)	0	13 (10.7)	1 (1.4)
Arthralgia	2 (1.5)	4 (3.1)	6 (5.5)	6 (7.4)	2 (1.5)	6 (4.8)	6 (5.0)	4 (5.6)
Peripheral neuropathy	10 (7.4)	1 (0.8)	0	1 (1.2)	5 (3.8)	0	2 (1.7)	4 (5.6)
Bone pain	0	0	8 (7.3)	0	1 (0.8)	3 (2.4)	6 (5.0)	0
Abdominal pain	8 (5.9)	2 (1.5)	2 (1.8)	1 (1.2)	6 (4.5)	1 (0.8)	3 (2.5)	3 (4.2)
Myalgia	1 (0.7)	3 (2.3)	4 (3.6)	4 (4.9)	2 (1.5)	4 (3.2)	6 (5.0)	7 (9.9)
Alopecia	2 (1.5)	2 (1.5)	5 (4.5)	2 (2.5)	3 (2.3)	5 (4.0)	13 (10.7)	4 (5.6)
Asthenia	6 (4.4)	7 (5.4)	3 (2.7)	2 (2.5)	9 (6.8)	3 (2.4)	1 (0.8)	1 (1.4)
Dizziness	2 (1.5)	2 (1.5)	5 (4.5)	3 (3.7)	2 (1.5)	2 (1.6)	7 (5.8)	0
Febrile neutropenia	1 (0.7)	1 (0.8)	5 (4.5)	2 (2.5)	0	3 (2.4)	7 (5.8)	0
Nausea	4 (3.0)	5 (3.8)	3 (2.7)	4 (4.9)	10 (7.6)	5 (4.0)	2 (1.7)	3 (4.2)
Paresthesia	6 (4.4)	0	0	0	8 (6.1)	0	0	1 (1.4)
Rash	2 (1.5)	1 (0.8)	4 (3.6)	1 (1.2)	0	3 (2.4)	6 (5.0)	0
Decreased neutrophil count	0	1 (0.8)	3 (2.7)	0	0	0	7 (5.8)	1 (1.4)
Stomatitis	3 (2.2)	1 (0.8)	1 (0.9)	1 (1.2)	2 (1.5)	1 (0.8)	10 (8.3)	0
Dyspnea	1 (0.7)	8 (6.2)	0	0	2 (1.5)	0	1 (0.8)	0
Musculoskeletal pain	0	1 (0.8)	3 (2.7)	1 (1.2)	0	3 (2.4)	5 (4.1)	7 (9.9)

*AE* adverse event, *ASaT* all-subjects-as-treated, *GI* gastrointestinal

^a^Grades 1 to 4 by maximum toxicity grade in subjects within either treatment arm

In the breast cancer subgroup, 1 serious treatment-related AE (hypersensitivity) was reported in the fosaprepitant regimen, which resolved after 30 min. Two serious treatment-related AEs (hypersensitivity and constipation) were also reported in the control group of the gynecologic cancer subgroup.

Ten subjects died during the study. Death was rare in both the fosaprepitant and control regimen treatment arms in the GI or colorectal (1.5 and 0%, respectively), lung (3.8 and 1.6%, respectively), breast (both 0%), and gynecologic (1.2 and 0%, respectively) cancer subgroups. All deaths during the study appeared to be attributable to subjects’ underlying malignancies, other preexisting conditions, and/or effects of chemotherapy, and none of the deaths were considered related to the study drug by the investigators.

## Discussion

The results of this post hoc analysis of the PN031 trial showed consistent efficacy of a single-day fosaprepitant regimen across common cancer subpopulations, including GI or colorectal, lung, breast, and gynecologic cancers. Compared with ondansetron plus dexamethasone alone (control regimen), the addition of fosaprepitant led to a greater proportion of subjects achieving CR across cancer subtypes in the delayed phase, with the largest treatment difference being observed in the subgroup of subjects with gynecologic cancer (22%). In the acute phase, both groups had good antiemetic control across cancer subgroups. Furthermore, time-to-first vomiting episode favored fosaprepitant across all cancer subgroups.

Cancer subgroup treatment response may be affected by chemotherapy type, chemotherapy duration, and predictive risk factors. In a recent publication outlining the development of a tool for the assessment of predictive risk factors of CINV, 8 risk factors were identified: patient age ≤ 60 years, the first cycle of chemotherapy, presence of anticipatory nausea and vomiting, a history of morning sickness, having fewer than 7 h of sleep the night before chemotherapy, CINV in the prior cycle, patient self-medication with nonprescribed antiemetics, and the use of platinum-based or AC-based regimens [18]. While all subjects were receiving non-AC MEC in the current analysis, the subjects in the gynecologic cancer subgroup were more commonly treated with carboplatin-based chemotherapy, which tends to have a higher risk of inducing CINV than noncarboplatin chemotherapy [6, 9], compared with the other cancer subtypes. This may explain, in part, why the largest treatment difference for CR (22%) was observed in this subgroup. The GI/colorectal cancer subgroup received more multiple-day than single-day chemotherapy regimens compared with the other cancer subgroups, which may have contributed to early divergence in time to vomiting in this cancer subgroup.

In an earlier exploratory analysis of this study, the effects of chemotherapy type (carboplatin-based vs. non–carboplatin-based) and chemotherapy duration (single-day vs. multiple-day) across all cancer subtypes were explored. That report found that the chemotherapy type and duration did not appear to influence response to fosaprepitant [19]. Whether subjects received either single- or multiple-day MEC regimens, or carboplatin-based or non–carboplatin-based chemotherapy regimens did not substantially affect CR rates with the fosaprepitant regimen, and CR was consistent (76–80%) during both the delayed and overall phases. However, compared with the control regimen, fosaprepitant exhibited more favorable treatment effects in the delayed phase of the carboplatin-based versus the non–carboplatin-based subgroup (14.1% vs. 6.5%), as well as for the single-day versus the multiple-day subgroup (13.2% vs. 3.2%). Fosaprepitant also exhibited more favorable treatment effects over control in the single-day versus the multiple-day treatment group in the overall phase (12.8% vs. 4.0%) [19]. This suggests that the treatment effect of the fosaprepitant regimen may be more pronounced in subjects receiving carboplatin-based and single-day chemotherapeutic regimens.

Previous studies have evaluated a range of cancer subpopulations in subjects receiving MEC and HEC (ie, including approximately 50% AC-based) regimens. Post hoc subanalyses of clinical trials investigating a rolapitant regimen (including a 5HT_3_RA and dexamethasone) evaluated CINV in specific cancer subtypes, including GI/colorectal, lung, and breast cancers [20, 21]. Their post hoc analysis of 84 subjects with GI or colorectal cancer who received MEC agents (predominantly irinotecan and oxaliplatin) reported higher CR rates among patients receiving a rolapitant regimen versus placebo in the acute (91.5% vs. 73.0%), delayed (74.5% vs. 54.1%), and overall phases (74.5% vs. 48.6%). In subjects with lung cancer (*n* = 687) receiving cisplatin or carboplatin, improvements in CR rates were seen with rolapitant versus an active control in the acute (88.4% vs. 81.7%), delayed (77.4% vs. 65.1%), and overall (75.4% vs. 63.1%) phases. Finally, in subjects with breast cancer (*n* = 845) who were receiving MEC or HEC (AC-based) regimens, CR rates were greater with rolapitant than with active control in the overall (62.8% vs. 55.1%) and delayed phases (66.7% vs. 59.8%) [20, 21]. While these post hoc analyses with rolapitant also support the antiemetic efficacy of a substance P/neurokinin-1 receptor antagonist for the prevention of CINV, the studies included a high proportion of HEC, including cisplatin and AC-based regimens, as well as carboplatin, whereas the PN031 study is a true MEC trial by current guideline definitions. However, the size of our current tumor type analysis of PN031 is smaller than the previously reported post hoc analyses of rolapitant studies, supporting the need for further investigation.

A pooled retrospective analysis of patient-level data from 4 large (*N* = 2813) randomized clinical trials of aprepitant was performed to characterize treatment response in subjects with various cancer subtypes (breast, GI, genitourinary, lung) who were receiving MEC and HEC (AC-based) regimens [22]. CR rates in subjects receiving MEC were higher for patients receiving aprepitant compared with those receiving the active control for all tumor types in the overall phase (0–120 h after chemotherapy initiation), with the largest difference noted among patients with breast cancer (54.9% vs. 43.9%). Although this retrospective analysis did not include a purely non-AC MEC population, the findings do further support the use of fosaprepitant in the MEC setting.

In the current analysis, the fosaprepitant regimen was generally well tolerated across all cancer subtypes. Serious treatment-related AEs were rare, and no deaths were considered related to the study drug. In general, the safety profile of the fosaprepitant regimen was consistent across all cancer subtypes, although AEs were most prevalent in the group with breast cancer, followed by GI or colorectal cancers. Most of the apparent differences between subgroups can be attributed to disease- or treatment-based toxicity, eg, nausea/vomiting and neutropenia in breast cancer [23] and nausea/vomiting and diarrhea in GI cancer [24].

Several study limitations should be considered when drawing conclusions from the results of the current analysis of PN031. Although the findings of this analysis support those of the primary study results [11], the current analysis was post hoc and exploratory. This analysis explored the efficacy and safety of the single-day fosaprepitant triple antiemetic regimen versus a 3-day active-control regimen in 4 tumor types. However, the tumor type comparisons are limited because of differences in antineoplastic agents and chemotherapy regimens, which may have varying degrees of emetogenic risk even within the context of MEC [4–6, 19]. Moreover, some cancer types that were represented in the trial population had sample sizes too small to analyze, among them head and neck cancer, germ cell cancer, hepatobiliary cancer, lymphoproliferative cancer, sarcoma, skin cancer, and other unspecified types. Finally, these findings should be interpreted with caution as sample sizes for some cancer subgroups were small.

## Conclusions

Findings of the current post hoc analysis of this phase III trial support those of the primary study—a single-day fosaprepitant regimen is effective in preventing CINV in subjects receiving non-AC-based MEC. The fosaprepitant regimen was effective across the most common cancer subpopulations in our study sample (GI/colorectal, lung, breast, gynecologic). Because the primary goal of antiemetic therapy is to prevent the occurrence of CINV, the consistent efficacy observed with a single dose of fosaprepitant across different tumor types is encouraging. As a result, adequately powered, randomized controlled studies prospectively evaluating these cancer subpopulations are warranted.

## Supplementary information

**Additional file 1.** List of Independent Ethics Committees (IECs).

## Data Availability

Merck Sharp & Dohme Corp., a subsidiary of Merck & Co., Inc., Kenilworth, NJ, USA’s data sharing policy, including restrictions, is available at http://engagezone.msd.com/ds_documentation.php. Requests for access to the clinical study data can be submitted through the EngageZone site or via email to dataaccess@merck.com.

## Abbreviations

AC: Anthracycline-cyclophosphamide
AE: Adverse event
ASaT: All subjects as treated
CI: Confidence interval
CINV: Chemotherapy-induced nausea and vomiting
CR: Complete response
GI: Gastrointestinal
HEC: Highly emetogenic chemotherapy
ITT: Intention-to-treat
IV: Intravenous
LEC: Low emetogenic chemotherapy
MEC: Moderately emetogenic chemotherapy
