# Supplementary material for: Single-dose fosaprepitant for the prevention of chemotherapy-induced nausea and vomiting in patients receiving moderately emetogenic chemotherapy regimens: a subgroup analysis from a randomized clinical trial of response in subjects by cancer type
Source: BMC Cancer. 2020 Sep 25;20:918. doi: 10.1186/s12885-020-07259-5 (PMC7523353; doi:10.1186/s12885-020-07259-5)
Supplement: Supplementary file 1 — Additional file 1. List of Independent Ethics Committees (IECs). [file 12885_2020_7259_MOESM1_ESM.docx]

**Additional File 1. List of Independent Ethics Committees (IECs)**

**Table.** List of IECs

| Site name | Location | IEC name |
| --- | --- | --- |
| Onkologiai Osztaly | Zalaegerszeg-Pozva, Hungary | ETT KFEB |
| Onkoterapias Intezet | Pecs, Hungary | ETT KFEB |
| Veterans Memorial Medical Center | Quezon City, Philippines | Veterans Memorial Medical Center IRB |
| Manila Doctor's Hospital | Manila, Philippines | Manila Doctor's Hospital IRB |
| Makati Medical Center | Makati City, Philippines | Makati Medical Center IRB |
| Ulleval Universitetssykehus HF | Oslo, Norway | REK soer-oest C |
| Avdeling for kreftbehandling, Kreft-, kirurgi- og transplantasjonsklinikken | Oslo, Norway | REK soer-oest C |
| Avdeling for blod- og kreftsykdommer | Oslo, Norway | REK soer-oest C |
| Hospital Provincial del Centenario | Rosario, Argentina | Comite de Docencia e Investigacion del Hospital Provincial del Centenario |
| Instituto Medico Especializado Alexander Fleming | Buenos Aires, Argentina | Comite Ejecutivo de Investigacion del Instituto Medico Especializado Alexander Fleming |
| COIBA | Buenos Aires, Argentina | Comite de Etica COIBA |
| Onkoradiologia | Gyor, Hungary | ETT KFEB |
| Onkoterapias Klinika | Szeged, Hungary | ETT KFEB |
| Deaconess Clinic | Evansville, IN, USA | Schulman Associates IRB |
| Comprehensive Cancer Center | Palm Springs, CA, USA | WIRB |
| University Medical Center New Orleans | New Orleans, LA, USA | Ochsner Clinic Foundation IRB |
| Billings Clinic Cancer Center | Billings, MT | Schulman Associates IRB |
| Eduardo G Gomez MD PA | Hialeah, FL | Schulman Associates IRB |
| Scott & White Healthcare Temple Clinic | Killeen, TX, USA | Scott & White IRB |
| Scott & White Medical Center – Temple | Killeen, TX, USA | Scott & White IRB |
| University of Colorado Health Cancer Care & Hematology | Ft. Collins, CO, USA | Poudre Valley Health System IRB |
| Scott & White Health Hematology Oncology | Round Rock, TX, USA | Scott & White IRB |
| Intermountain Healthcare | Seattle, WA, USA | Quorum Review IRB |
| Lapidus Cancer Institute | Baltimore, MD, USA | MedStar Health IRB |
| MedStar Franklin Square Cancer Center | Baltimore, MD, USA | MedStar Health IRB |
| Lakeland Regional Cancer Center | Lakeland, FL, USA | Lakeland Regional Cancer Center IRB |
| Maryland Oncology Hematology | Rockville, MD, USA | Schulman Associates IRB |
| Physicians' Clinic of Iowa | Cedar Rapids, IA, USA | Schulman Associates IRB |
| UOC Oncologia Medica | Benevento, Italy | Azienda Ospedalera G. Rummo |
| Hospital de la Fuerza Aerea del Peru | Lima, Peru | Comite de Etica en Investigación del Hospital de la Fuerza Aerea del Peru |
| Red Asistencial AQP | Lima, Peru | Comite de Etica en Investigacion de la Red Asistencial AQP |
| Hospital Nacional Almanzor Aguinaga Asenjo | Lima, Peru | Comite de Etica Investigacion del Hospital Nacional Almanzor Aguinaga Asenjo |
| RAKUS (2 sites) | Riga, Latvia | RAKUS Atbalsta f. Med. un biomed. petijumu Etikas komiteja |
| University Hospital Center Sestre Milosrdnice | Zagreb, Croatia | Središnje etičko povjerenstvo |
| University Hospital for Tumors, Sestre milosrdnice University Hospital Center | Zagreb, Croatia | Središnje etičko povjerenstvo |
| Erasmus MC Cancer Care | Breda, Netherlands | Stichting Beoordeling Ethiek Biomedisch Onderzoek |
| Spaarne Gasthuis | Hoofddorp, Netherlands | Stichting Beoordeling Ethiek Biomedisch Onderzoek |
| Zuyderland Medical Center | Sittard-Geleen, Netherlands | Stichting Beoordeling Ethiek Biomedisch Onderzoek |
| Albert Schweitzer Hospital | Dordrecht, Netherlands | Stichting Beoordeling Ethiek Biomedisch Onderzoek |
| VieCuri Hospital Venlo/Venray | Venray, Netherlands | Stichting Beoordeling Ethiek Biomedisch Onderzoek |
| Vaasa Oncology Clinic | Vaasa, Finland | TUKIJA |
| Kuopio University Hospital | Kuopio, Finland | TUKIJA |
| Azienda Ospedaliera Santa Maria | Terni, Italy | CE delle Aziende Sanitarie dell'Umbria |
| Ospedale Civile SS Annunziata | Sassari, Italy | C.E. Della ASL di Sassari |
| Uppsala University | Uppsala, Sweden | Regionala etikprovningsnamnden Uppsala |
| Karolinska University Hospital | Stockholm, Sweden | Regionala etikprovningsnamnden Uppsala |
| Sahlgrenska University Hospital (2 sites) | Gothenburg, Sweden | Regionala etikprovningsnamnden Uppsala |
| HPTU | Medellin, Colombia | Comite de Investigaciones y Etica en Investigaciones HPTU |
| FSFB | Bogota, Colombia | Comite Corporativo de Etica en Investigacion FSFB |
| Clinica del Country | Bogota, Colombia | Comite de Etica en Investigacion Clinica - Clinica del Country |
| IMAT | Monteria, Colombia | Comite De Etica E Investigaciones |
| MHAT | Sofia, Bulgaria | EC for Multicenter Trials |
| Complex Oncological Center | Ruse, Bulgaria | EC for Multicenter Trials |
| Hospital ISSSTE Presidente Juárez | Oaxaca, Mexico | Oaxaca Site Management Organization SC |
| USMP | Cuzco, Peru | Comité Institucional de Etica en Inv USMP - Clin. CadaMujer |
| Gaziantep University Faculty of Medicine | Gaziantep, Turkey | Gaziantep University Faculty of Medicine EC |
| Istanbul University-Cerrahpasa | Istanbul, Turkey | Gaziantep University Faculty of Medicine EC |
| Adana City Training & Research Hospital | Adana, Turkey | Gaziantep University Faculty of Medicine EC |
| N.N. Blokhin Cancer Research Center (2) | Moscow, Russia | IIEC on Ethical Review for Clinical Studies |
| Ev. Huyssens-Stiftung/Knappschaft GmbH, Kliniken Essen-Mitte | Essen, Germany | Ethik-Kommission der Medizinischen Fakultaet |
| Klinik fuer Haematologie/Onkologie | Eschweiler, Germany | Ethik-Kommission der Medizinischen Fakultaet |
| Onkologische Praxis Oldenburg | Oldenburg, Germany | Ethik-Kommission der Medizinischen Fakultaet |
| University of Heidelberg | Heidelberg, Germany | Ethik-Kommission der Medizinischen Fakultaet |
| Uniklinik u.Poliklinik f.Innere Medizin IV | Halle, Germany | Ethik-Kommission der Medizinischen Fakultaet |
| Klinik für Innere Medizin - Hämatologie, Onkologie, Rheumatologie und Palliativmedizin | Berlin, Germany | Ethik-Kommission der Medizinischen Fakultaet |
| Kharikiv Regional Clinical Oncology Center | Kharkiv, Ukraine | LEC of Kharikiv Regional Clinical Oncology Center |
| Dnipropetrovsk City Multidiscipline Clinical Hospital | Dnipropetrovsk, Ukraine | LEC of Dnipropetrovsk City Multidiscipline Clinical Hospital |
| SANADOR Clinical Hospital | Bucharest, Romania | Comisia Nationala de Etica pentru Studiul Clinic al Medicamentului |
| Sp. Univ. de Urg. Buc. | Bucharest, Romania | Comisia Nationala de Etica pentru Studiul Clinic al  Medicamentului |
| Institutul Oncologic Bucuresti | Bucharest, Romania | Comisia Nationala de Etica pentru Studiul Clinic al  Medicamentului |
| S.C. Medicover Romania S.R.L | Ploiesti, Romania | Comisia Nationala de Etica pentru Studiul Clinic al  Medicamentului |
| Bucharest Oncology Institute | Bucharest, Romania | Comisia Nationala de Etica pentru Studiul Clinic al  Medicamentului |
| Moscow Research Oncological Institute n.a. P.A. Herzen | Moscow, Russia | Ethics Board under the Ministry of Health of the Russian Federation |
| Vall d'Hebron Hospital | Barcelona, Spain | CEIC Hospitals Vall d'Hebron |
| H G U de Elche | Alicante, Spain | CEIC Hospitals Vall d'Hebron |
| Hospital Arnau de Vilanova | Leloida, Spain | CEIC Hospitals Vall d'Hebron |
| Hospital Clinico San Carlos | Madrid, Spain | CEIC Hospitals Vall d'Hebron |
| Hospital Miguel Servet | Zaragoza, Spain | CEIC Hospitals Vall d'Hebron |
| Regional Oncology Dispensary Medgorodok | Chelyabinsk, Russia | Chelyabinsk City Oncology Health Centre |
| Centro Hospitalar Lisboa Norte - Hospital de Santa Maria | Lisbon, Portugal | CEIC - Comissao de Etica para a Investigacao Clinica |
| University Hospital of Heraklion | Heraklion, Greece | PAGNH |
| Instituto Português de Oncologia do Porto Francisco Gentil | Porto, Portugal | CEIC - Comissao de Etica para a Investigacao Clinica |
| Gerasimos Aravantinos | Athens, Greece | EC of Gen. Onc. Hosp. of Kifisia “Agioi Anargyroi” |
| Metropolitan Hospital | Athens, Greece | Hippokrateion General Hospital of Athens |
| Hôpital du Sacré-Cœur de Montréal | Montreal, QC, Canada | Hospital du Sacre-Coeur de Montreal IRB |
| Voivodeship Specialized Hospital No. 3 | Rybnik, Poland | Komisja Bioetyczna Okregowej Izby Lekarskiej W |
| Medical University of Lublin | Lublin, Poland | Komisja Bioetyczna Okregowej Izby Lekarskiej W |
| Magodent | Warsaw, Poland | Komisja Bioetyczna Okregowej Izby Lekarskiej W |
| Chulalongkorn University and the King Chulalongkorn Memorial Hospital | Bangkok, Thailand | IRB Faculty of Medicine, Chulalongkorn University |
| Krakowski Szpital Specjalistyczny im. Jana Pawła II | Krakow, Poland | Komisja Bioetyczna Okregowej Izby Lekarskiej W |
| Oncology Center | Krakow, Poland | Komisja Bioetyczna Okregowej Izby Lekarskiej W |
| University Hospital Motol | Prague, Czech Republic | Fakultni Nemocnice Kralovske Vinohrady |
| LF UK a FN Královské Vinohrady | Prague, Czech Republic | Fakultni Nemocnice Kralovske Vinohrady |
| City Hospital | Usti nad Labem, Czech Republic | Fakultni Nemocnice Kralovske Vinohrady |
| Faculty Hospital Hradec Králové | Hradec Králové, Czech Republic | Fakultni Nemocnice Kralovske Vinohrady |
| Sandton Oncology Centre | Johannesburg, South Africa | Pharma Ethics |
| Ramon y Cajal University Hospital, Ctra | Madrid, Spain | CEIC Hospitals Vall d'Hebron |
| Hospital de la Santa Creu i Sant Pau | Barcelona, Spain | CEIC Hospitals Vall d'Hebron |
| Carle Cancer Center | Urbana, IL, USA | Schulman Associates IRB |
| Jupiter Medical Center | Jupiter, FL, USA | Jupiter Medical Center IRB |
| St. Vincent Hospital Cancer & Wellness Ctr | Worcester, MA, USA | IRB Saint Vincent Hospital |
| Oncology Consultants | Houston, TX, USA | BRANY |
| University of South Alabama | Mobile, AL, USA | University of South Alabama IRB |
| Asheville VA Medical Center | Asheville, NC, USA | Asheville VA Medical Center IRB no. 1 |
| Charleston Oncology | Charleston, SC, USA | Schulman Associates IRB |
| Florida Cancer Center | Boynton Beach, FL, USA | Schulman Associates IRB |
| Costal Bend Cancer Center | Corpus Christi, TX, USA | Schulman Associates IRB |
| Hospital Militar de Santiago | Santiago, Chile | Comite Etica Cientifico Servicio de SSMO |
| Arturo Lopez Foundation, Institute of Oncology | Santiago, Chile | Comite Etica Cientifico Servicio de SSMO |
| Charles University | Prague, Czech Republic | Fakultni Nemocnice Kralovske Vinohrady |
| Horovice Oncology Clinic, Nemocnice Rudolfa a Stefanie Benešov, a. s | Prague, Czech Republic | Fakultni Nemocnice Kralovske Vinohrady |
| Comprehensive Oncology Center | Milada Boleslav, Czech Republic | Fakultni Nemocnice Kralovske Vinohrady |
| Nemocnice Na Bulovce | Prague, Czech Republic | Fakultni Nemocnice Kralovske Vinohrady |
| 401 Army General Hospital | Athens, Greece | IASO General |
| Policlinica Oncomed Timisoara | Timisoara, Romania | Comisia Nationala de Etica pentru Studiul Clinic al Medicamentului |
| Onkologkliniken Sormland | Eskilstuna, Sweden | Regionala etikprovningsnamnden Uppsala |
| Kocaeli UTF Enfeksiyon Hastaliklari BD. | Gaziantep, Turkey | Gaziantep University Faculty of Medicine EC |
| Kocaeli University Medical Faculty Hospital | Kocaeli, Turkey | Gaziantep University Faculty of Medicine EC |
| Akdeniz University Hospital | Antalya, Turkey | Gaziantep University Faculty of Medicine EC |
| Mercy Health-Paducah Medical Oncology and Hematology | Paducah, KY, USA | Schulman Associates IRB |
| Comprehensive Cancer Care and Research Institute of Colorado | Englewood, CO, USA | Schulman Associates IRB |
| Hematology/Oncology Associates of Treasure Coast | Port St. Lucie, FL, USA | Schulman Associates IRB |
| Northern Utah Associates | Ogden, UT, USA | Schulman Associates IRB |
| Baptist Health Medical Group | Lexington, KY, USA | Baptist Healthcare System, Central Baptist Hospital IRB |
| Lakes Research | Miami Lakes, FL, USA | Schulman Associates IRB |
| Mercy Clinic Cancer and Hematology | Springfield, MO, USA | Mercy Health Springfield Communities IRB |
| Universidad Autónoma de Nuevo León | Monterrey, Mexico | Comite de Etica en Investigacion del Hospital Santa Cecilia de Monterrey S.A. de C.V. |
| Metropolitan Oncology Center Psc | San Juan, Puerto Rico | Schulman Associates IRB |
| FDI Clinical Research | San Juan, Puerto Rico | Schulman Associates IRB |
| Ponce Hematology Oncology | Ponce, Puerto Rico | Schulman Associates IRB |
| McGill University Health Centre | Montreal, QC, Canada | McGill University Faculty of Medicine IRB |
| Carmen Carolina Tokumura Tokumura | Lima, Peru | Comite Institucional Etica en Investigacion - Hospital Nacional Cayetano Heredia |
| City Cancer Hospital | St. Petersburg, Russia | Ethics Board under the Ministry of Health of the Russian Federation |
| Mid Ohio Oncology/Hematology | Columbus, OH, USA | Schulman Associates IRB |
| Community Clinical Research Center | Anderson, IN, USA | Schulman Associates IRB |
| MedStar Harbor Hospital | Baltimore, MD, USA | Georgetown University IRB |
| SA Medical Specialists & Health Practitioners | Durban, South Africa | Pharma Ethics |
| Medical Oncology Centre of Rosebank | Johannesburg, South Africa | Pharma Ethics |
| The Mary Potter Oncology Centre | Pretoria, South Africa | Pharma Ethics |
| Wilgers Oncology Centre | Pretoria, South Africa | Pharma Ethics |
| Northwest Medical Specialties, Inc. | Tacoma, WA, USA | Schulman Associates IRB |

EC, ethics committee; ETT KFEB, Egeszsegugyi Tudomanyos Tanacs, Klinikai Farmakologiai Etikai Bizottsaga; IRB, institutional review board; LEC, local ethics committee; SSMO, Salud Metropolitano Oriente; WIRB, Western Institutional Review Board.
